# Methyl 2,3-(3,6,9-trioxaundecane-1,11-diyldithio)-1,4,5,8-tetra­thia­fulvalene-6-carboxyl­ate

**DOI:** 10.1107/S1600536809013154

**Published:** 2009-04-18

**Authors:** Rui-bin Hou, Bao Li, Bing-zhu Yin, Li-xin Wu

**Affiliations:** a Key Laboratory of Organism Functional Factors of Changbai Mountain, Yanbian University, Ministry of Education, Yanji 133002, People’s Republic of China; b State Key Laboratory of Supramolecular Structures and Materials, College of Chemistry, Jilin University, Changchun 130012, People’s Republic of China

## Abstract

In the title mol­ecule, C_16_H_20_O_5_S_6_, the two five-membered rings form a dihedral angle of 4.7 (3)°. The crystal packing exhibits weak inter­molecular C—H⋯O hydrogen bonds, which link the mol­ecules into chains propagating in [1
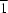
0], and π–π inter­actions, indicated by the short distances [3.756 (5) Å] between the centroids of five-membered rings from mol­ecules related by translation along the *b* axis.

## Related literature

For background to tetra­thia­fulvalene derivatives, see Hansen *et al.* (1992 ▶); Trippé *et al.* (2002 ▶). For details of the synthesis, see Liu *et al.* (2000 ▶).
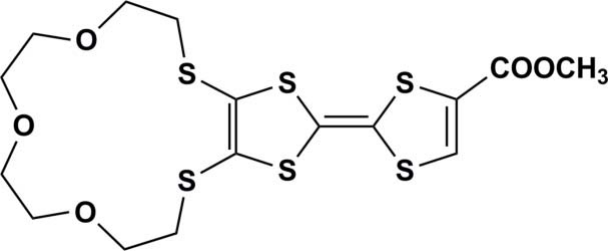


## Experimental

### 

#### Crystal data

C_16_H_20_O_5_S_6_
*M* *_r_* = 484.68
Monoclinic, 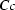

*a* = 22.604 (5) Å
*b* = 5.2048 (10) Å
*c* = 17.801 (4) Å
β = 90.65 (3)°
*V* = 2094.1 (7) Å^3^
*Z* = 4
Mo *K*α radiation
μ = 0.68 mm^−1^
*T* = 291 K
0.20 × 0.13 × 0.12 mm

#### Data collection

Rigaku R-AXIS RAPID diffractometer
Absorption correction: multi-scan (*ABSCOR*; Higashi, 1995 ▶) *T*_min_ = 0.876, *T*_max_ = 0.923
9505 measured reflections
4254 independent reflections
3370 reflections with *I* > 2σ(*I*)
*R*_int_ = 0.101

#### Refinement

*R*[*F* ^2^ > 2σ(*F* ^2^)] = 0.060
*wR*(*F* ^2^) = 0.172
*S* = 0.96
4254 reflections
245 parameters
2 restraints
H-atom parameters constrained
Δρ_max_ = 0.49 e Å^−3^
Δρ_min_ = −0.46 e Å^−3^
Absolute structure: Flack (1983 ▶), 1855 Friedel pairs
Flack parameter: −0.12 (12)

### 

Data collection: *RAPID-AUTO* (Rigaku, 1998 ▶); cell refinement: *RAPID-AUTO*; data reduction: *CrystalStructure* (Rigaku/MSC, 2002 ▶); program(s) used to solve structure: *SHELXS97* (Sheldrick, 2008 ▶); program(s) used to refine structure: *SHELXL97* (Sheldrick, 2008 ▶); molecular graphics: *PLATON* (Spek, 2009 ▶); software used to prepare material for publication: *SHELXL97*.

## Supplementary Material

Crystal structure: contains datablocks global, I. DOI: 10.1107/S1600536809013154/cv2536sup1.cif

Structure factors: contains datablocks I. DOI: 10.1107/S1600536809013154/cv2536Isup2.hkl

Additional supplementary materials:  crystallographic information; 3D view; checkCIF report

## Figures and Tables

**Table 1:** Hydrogen-bond geometry (Å, °)

*D*—H⋯*A*	*D*—H	H⋯*A*	*D*⋯*A*	*D*—H⋯*A*
C4—H4⋯O3^i^	0.93	2.35	3.127 (7)	141

Symmetry code: (i) 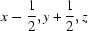.

**Fig. 1.**
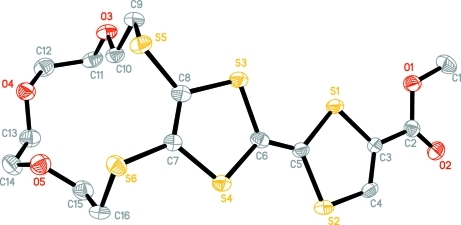
The molecular structure of (I) with the atom numbering. Displacement ellipsoids are drawn at the 30% probalility level. H atoms omitted for clarity.

## supplementary crystallographic information

### Comment

Cation sensors based on tetrathiafulvalene (TTF) derivatives have currently attracted widespread attention because such molecules show electrochemical recognition of various metal cations (Trippé *et al.*, 2002). We incorporated TTF with a 15-membered O, S hybrid crown ether to synthesize the title compound because it should be able to bind lithium ion (Hansen *et al.*, 1992). We report herein the synthesis and structure of the title compound, (I).

The molecular structure of (I),*C*_16_H_20_O_5_S_6_, is shown in Fig.1. Every molecule contains one TTF moiety and one dithia-15-crown-5 ring.TTF moiety is composed of two nearly coplanar five-membered rings with a dehedral angle of 4.68 (27) °. The dithia-15-crown-5 ring adopt a twiste conformation and situated almost perpendicular to TTF moiety. Owing to the absence of strong hydrogen bond donors, the crystal packing is stabilized by weak C—H···O hydrogen bonds, involving the O atoms of the crown ether as acceptors, and the methyl C—H groups as donors (Table 1). The crystal packing exhibits also π–π interactions, proved by short distance Cg1···Cg2^ii^ of 3.756 (5) Å, where Cg1 and Cg2 are centroids of S1/C3/C4/S2/C5 and S3/C6/S4/C7/C8 rings, respectively [symmetry code: (ii) x, 1+y, z].

### Experimental

6,7-Dimethoxycarbonyl-2,3- bis(3',6',9'-trioxoundecylthio)-1,4,5,8-tetrathiafulvalene (Liu *et al.*, 2000) (500 mg, 0.92 mmol) were dissovled in DMF (40 ml), LiBr (0.91 g, 10.5 mmol) and a drop of water was added. The mixture was heated at 80 °C for 2 h. After cooling to room temperature, saturated aqueous sodium chloride was added, and the mixture was extracted with ethyl acetate. The organic layer was washed with water, dried (MgSO_4_) and then concentrated under reduced pressure. The resulting red oil was purified by column chromatography [silica gel, eluent CH_2_Cl_2_-AcOEt (4: 1 *v*/*v*)] to afford the title compound as a red solid. Single crystals suitable for X-ray diffraction were prepared by slow evaporation of dichloromethane-n-hexane solution at room temperature.

### Refinement

C-bound H-atoms were placed in calculated positions (C—H 0.93-0.97 Å) and were included in the refinement in the riding model, with *U*_iso_(H) = 1.2 or 1.5 *U*_eq_(C).

### Crystal data

**Table:**

C_16_H_20_O_5_S_6_	*F*(000) = 1008
*M**_r_* = 484.68	*D*_x_ = 1.537 Mg m^−^^3^
Monoclinic, *C**c*	Mo *K*α radiation, λ = 0.71073 Å
Hall symbol: C -2yc	Cell parameters from 7343 reflections
*a* = 22.604 (5) Å	θ = 3.4–27.1°
*b* = 5.2048 (10) Å	µ = 0.68 mm^−^^1^
*c* = 17.801 (4) Å	*T* = 291 K
β = 90.65 (3)°	Block, red
*V* = 2094.1 (7) Å^3^	0.20 × 0.13 × 0.12 mm
*Z* = 4	

### Data collection

**Table:**

Rigaku R-AXIS RAPID diffractometer	4254 independent reflections
Radiation source: fine-focus sealed tube	3370 reflections with *I* > 2σ(*I*)
graphite	*R*_int_ = 0.101
ω scans	θ_max_ = 27.5°, θ_min_ = 3.6°
Absorption correction: multi-scan (*ABSCOR*; Higashi, 1995)	*h* = −28→28
*T*_min_ = 0.876, *T*_max_ = 0.923	*k* = −6→6
9505 measured reflections	*l* = −23→23

### Refinement

**Table:**

Refinement on *F*^2^	Secondary atom site location: difference Fourier map
Least-squares matrix: full	Hydrogen site location: inferred from neighbouring sites
*R*[*F*^2^ > 2σ(*F*^2^)] = 0.060	H-atom parameters constrained
*wR*(*F*^2^) = 0.172	*w* = 1/[σ^2^(*F*_o_^2^) + (0.0878*P*)^2^] where *P* = (*F*_o_^2^ + 2*F*_c_^2^)/3
*S* = 0.96	(Δ/σ)_max_ = 0.001
4254 reflections	Δρ_max_ = 0.49 e Å^−^^3^
245 parameters	Δρ_min_ = −0.45 e Å^−^^3^
2 restraints	Absolute structure: Flack (1983), 1855 Friedel pairs
Primary atom site location: structure-invariant direct methods	Flack parameter: −0.12 (12)

### Special details

**Table:**

Experimental. (See detailed section in the paper)
Geometry. All e.s.d.'s (except the e.s.d. in the dihedral angle between two l.s. planes) are estimated using the full covariance matrix. The cell e.s.d.'s are taken into account individually in the estimation of e.s.d.'s in distances, angles and torsion angles; correlations between e.s.d.'s in cell parameters are only used when they are defined by crystal symmetry. An approximate (isotropic) treatment of cell e.s.d.'s is used for estimating e.s.d.'s involving l.s. planes.
Refinement. Refinement of *F*^2^ against ALL reflections. The weighted *R*-factor *wR* and goodness of fit *S* are based on *F*^2^, conventional *R*-factors *R* are based on *F*, with *F* set to zero for negative *F*^2^. The threshold expression of *F*^2^ > σ(*F*^2^) is used only for calculating *R*-factors(gt) *etc*. and is not relevant to the choice of reflections for refinement. *R*-factors based on *F*^2^ are statistically about twice as large as those based on *F*, and *R*- factors based on ALL data will be even larger.

### Fractional atomic coordinates and isotropic or equivalent isotropic displacement parameters (Å^2^)

**Table:**

	*x*	*y*	*z*	*U*_iso_*/*U*_eq_	
C1	0.4826 (4)	1.8931 (13)	0.5758 (4)	0.0638 (17)	
H1A	0.4419	1.8858	0.5598	0.096*	
H1B	0.5076	1.8546	0.5340	0.096*	
H1C	0.4915	2.0622	0.5942	0.096*	
C2	0.4534 (3)	1.7154 (11)	0.6912 (3)	0.0426 (12)	
C3	0.4679 (2)	1.5069 (10)	0.7458 (3)	0.0388 (11)	
C4	0.4324 (2)	1.4479 (11)	0.8031 (3)	0.0413 (11)	
H4	0.3979	1.5404	0.8115	0.050*	
C5	0.5199 (2)	1.1345 (9)	0.8157 (3)	0.0368 (10)	
C6	0.5581 (2)	0.9558 (10)	0.8382 (3)	0.0392 (11)	
C7	0.6165 (2)	0.6004 (9)	0.9111 (3)	0.0361 (10)	
C8	0.6520 (2)	0.6615 (10)	0.8524 (3)	0.0405 (11)	
C9	0.7699 (3)	0.7676 (12)	0.8282 (3)	0.0507 (13)	
H9A	0.8086	0.7011	0.8154	0.061*	
H9B	0.7580	0.8888	0.7895	0.061*	
C10	0.7740 (3)	0.9042 (10)	0.9022 (3)	0.0438 (12)	
H10A	0.7372	0.9930	0.9122	0.053*	
H10B	0.7813	0.7818	0.9423	0.053*	
C11	0.8288 (3)	1.2375 (11)	0.9635 (4)	0.0546 (15)	
H11A	0.7901	1.2783	0.9834	0.066*	
H11B	0.8474	1.3978	0.9492	0.066*	
C12	0.8653 (3)	1.1139 (13)	1.0241 (4)	0.0609 (16)	
H12A	0.9037	1.0707	1.0039	0.073*	
H12B	0.8715	1.2373	1.0643	0.073*	
C13	0.7993 (3)	0.9435 (13)	1.1131 (4)	0.0578 (15)	
H13A	0.7692	1.0616	1.0949	0.069*	
H13B	0.8202	1.0245	1.1547	0.069*	
C14	0.7710 (3)	0.7016 (13)	1.1390 (4)	0.0606 (16)	
H14A	0.8020	0.5776	1.1497	0.073*	
H14B	0.7511	0.7368	1.1858	0.073*	
C15	0.6760 (3)	0.7176 (13)	1.0822 (3)	0.0577 (16)	
H15A	0.6784	0.8429	1.0420	0.069*	
H15B	0.6677	0.8086	1.1284	0.069*	
C16	0.6266 (3)	0.5309 (13)	1.0653 (3)	0.0519 (14)	
H16A	0.5894	0.6240	1.0649	0.062*	
H16B	0.6250	0.4058	1.1056	0.062*	
O1	0.49274 (19)	1.7087 (8)	0.6344 (2)	0.0524 (10)	
O2	0.4141 (2)	1.8633 (9)	0.6967 (2)	0.0571 (11)	
O3	0.82120 (18)	1.0828 (8)	0.8984 (2)	0.0526 (10)	
O4	0.8395 (2)	0.8877 (8)	1.0548 (2)	0.0560 (10)	
O5	0.7302 (2)	0.5875 (8)	1.0894 (2)	0.0587 (11)	
S1	0.53276 (5)	1.3254 (3)	0.73569 (7)	0.0415 (3)	
S2	0.45240 (6)	1.1969 (3)	0.86052 (7)	0.0456 (3)	
S3	0.62439 (6)	0.8944 (3)	0.78907 (7)	0.0453 (3)	
S4	0.54731 (6)	0.7502 (3)	0.91476 (7)	0.0417 (3)	
S5	0.71732 (7)	0.5055 (3)	0.83051 (8)	0.0492 (4)	
S6	0.63337 (7)	0.3600 (3)	0.97634 (7)	0.0486 (4)	

### Atomic displacement parameters (Å^2^)

**Table:**

	*U*^11^	*U*^22^	*U*^33^	*U*^12^	*U*^13^	*U*^23^
C1	0.081 (5)	0.063 (4)	0.047 (3)	−0.001 (4)	0.006 (3)	0.016 (3)
C2	0.043 (3)	0.044 (3)	0.040 (3)	−0.004 (2)	−0.003 (2)	−0.004 (2)
C3	0.031 (3)	0.049 (3)	0.036 (3)	0.000 (2)	0.0000 (19)	0.000 (2)
C4	0.028 (3)	0.050 (3)	0.046 (3)	−0.001 (2)	0.005 (2)	−0.005 (2)
C5	0.032 (3)	0.042 (2)	0.037 (3)	−0.002 (2)	0.0045 (19)	−0.0003 (19)
C6	0.044 (3)	0.038 (2)	0.035 (2)	−0.013 (2)	0.005 (2)	−0.0006 (18)
C7	0.038 (3)	0.033 (2)	0.038 (2)	−0.005 (2)	0.002 (2)	−0.0025 (18)
C8	0.045 (3)	0.038 (2)	0.038 (3)	−0.006 (2)	0.006 (2)	−0.0047 (19)
C9	0.046 (3)	0.064 (3)	0.042 (3)	0.000 (3)	0.014 (2)	0.004 (2)
C10	0.039 (3)	0.044 (3)	0.049 (3)	−0.002 (2)	0.011 (2)	0.003 (2)
C11	0.048 (3)	0.043 (3)	0.074 (4)	−0.003 (3)	0.021 (3)	−0.001 (3)
C12	0.049 (4)	0.062 (4)	0.072 (4)	−0.008 (3)	0.008 (3)	0.001 (3)
C13	0.058 (4)	0.063 (4)	0.053 (3)	−0.005 (3)	0.007 (3)	−0.009 (3)
C14	0.065 (4)	0.070 (4)	0.047 (3)	−0.009 (3)	−0.003 (3)	0.007 (3)
C15	0.071 (4)	0.061 (3)	0.042 (3)	0.011 (3)	0.006 (3)	−0.006 (3)
C16	0.048 (3)	0.071 (4)	0.038 (3)	0.003 (3)	0.018 (2)	0.006 (2)
O1	0.050 (2)	0.060 (2)	0.048 (2)	0.014 (2)	0.0091 (18)	0.0153 (18)
O2	0.050 (2)	0.064 (2)	0.058 (3)	0.024 (2)	0.0010 (19)	0.0008 (19)
O3	0.043 (2)	0.058 (2)	0.057 (2)	−0.007 (2)	0.0155 (18)	0.0001 (18)
O4	0.061 (3)	0.052 (2)	0.054 (2)	0.006 (2)	0.0100 (19)	0.0062 (17)
O5	0.056 (3)	0.062 (2)	0.058 (3)	−0.001 (2)	0.001 (2)	−0.0022 (19)
S1	0.0371 (7)	0.0476 (7)	0.0400 (6)	0.0027 (6)	0.0104 (5)	0.0065 (5)
S2	0.0393 (7)	0.0540 (7)	0.0439 (7)	−0.0046 (6)	0.0138 (5)	0.0040 (6)
S3	0.0413 (7)	0.0513 (7)	0.0436 (7)	0.0017 (6)	0.0109 (5)	0.0095 (5)
S4	0.0392 (7)	0.0459 (7)	0.0403 (6)	−0.0028 (5)	0.0083 (5)	0.0052 (5)
S5	0.0482 (8)	0.0440 (7)	0.0557 (9)	0.0045 (6)	0.0117 (6)	−0.0058 (6)
S6	0.0592 (9)	0.0414 (7)	0.0452 (7)	−0.0013 (6)	0.0026 (6)	0.0061 (5)

### Geometric parameters (Å, °)

**Table:**

C1—O1	1.434 (7)	C10—O3	1.418 (7)
C1—H1A	0.9600	C10—H10A	0.9700
C1—H1B	0.9600	C10—H10B	0.9700
C1—H1C	0.9600	C11—O3	1.420 (7)
C2—O2	1.182 (7)	C11—C12	1.496 (10)
C2—O1	1.354 (7)	C11—H11A	0.9700
C2—C3	1.491 (7)	C11—H11B	0.9700
C3—C4	1.342 (7)	C12—O4	1.425 (8)
C3—S1	1.754 (5)	C12—H12A	0.9700
C4—S2	1.716 (6)	C12—H12B	0.9700
C4—H4	0.9300	C13—O4	1.418 (7)
C5—C6	1.328 (7)	C13—C14	1.487 (9)
C5—S2	1.761 (5)	C13—H13A	0.9700
C5—S1	1.763 (5)	C13—H13B	0.9700
C6—S4	1.752 (5)	C14—O5	1.403 (8)
C6—S3	1.772 (6)	C14—H14A	0.9700
C7—C8	1.362 (7)	C14—H14B	0.9700
C7—S6	1.747 (5)	C15—O5	1.404 (8)
C7—S4	1.749 (6)	C15—C16	1.507 (10)
C8—S5	1.733 (6)	C15—H15A	0.9700
C8—S3	1.765 (6)	C15—H15B	0.9700
C9—C10	1.498 (8)	C16—S6	1.824 (6)
C9—S5	1.811 (6)	C16—H16A	0.9700
C9—H9A	0.9700	C16—H16B	0.9700
C9—H9B	0.9700		
			
O1—C1—H1A	109.5	C12—C11—H11B	108.8
O1—C1—H1B	109.5	H11A—C11—H11B	107.7
H1A—C1—H1B	109.5	O4—C12—C11	114.1 (5)
O1—C1—H1C	109.5	O4—C12—H12A	108.7
H1A—C1—H1C	109.5	C11—C12—H12A	108.7
H1B—C1—H1C	109.5	O4—C12—H12B	108.7
O2—C2—O1	125.4 (5)	C11—C12—H12B	108.7
O2—C2—C3	125.5 (6)	H12A—C12—H12B	107.6
O1—C2—C3	109.1 (5)	O4—C13—C14	109.6 (5)
C4—C3—C2	122.2 (5)	O4—C13—H13A	109.8
C4—C3—S1	117.6 (4)	C14—C13—H13A	109.8
C2—C3—S1	120.2 (4)	O4—C13—H13B	109.8
C3—C4—S2	118.1 (4)	C14—C13—H13B	109.8
C3—C4—H4	121.0	H13A—C13—H13B	108.2
S2—C4—H4	121.0	O5—C14—C13	116.4 (5)
C6—C5—S2	123.8 (4)	O5—C14—H14A	108.2
C6—C5—S1	121.7 (4)	C13—C14—H14A	108.2
S2—C5—S1	114.4 (3)	O5—C14—H14B	108.2
C5—C6—S4	124.5 (4)	C13—C14—H14B	108.2
C5—C6—S3	121.9 (4)	H14A—C14—H14B	107.3
S4—C6—S3	113.6 (3)	O5—C15—C16	110.5 (5)
C8—C7—S6	123.4 (4)	O5—C15—H15A	109.5
C8—C7—S4	117.4 (4)	C16—C15—H15A	109.5
S6—C7—S4	118.9 (3)	O5—C15—H15B	109.5
C7—C8—S5	125.0 (4)	C16—C15—H15B	109.5
C7—C8—S3	116.3 (4)	H15A—C15—H15B	108.1
S5—C8—S3	118.3 (3)	C15—C16—S6	114.7 (4)
C10—C9—S5	111.8 (4)	C15—C16—H16A	108.6
C10—C9—H9A	109.3	S6—C16—H16A	108.6
S5—C9—H9A	109.3	C15—C16—H16B	108.6
C10—C9—H9B	109.3	S6—C16—H16B	108.6
S5—C9—H9B	109.3	H16A—C16—H16B	107.6
H9A—C9—H9B	107.9	C2—O1—C1	115.2 (5)
O3—C10—C9	107.9 (4)	C10—O3—C11	114.6 (4)
O3—C10—H10A	110.1	C13—O4—C12	112.3 (5)
C9—C10—H10A	110.1	C14—O5—C15	114.9 (5)
O3—C10—H10B	110.1	C3—S1—C5	94.3 (2)
C9—C10—H10B	110.1	C4—S2—C5	95.4 (2)
H10A—C10—H10B	108.4	C8—S3—C6	95.9 (3)
O3—C11—C12	113.9 (5)	C7—S4—C6	96.3 (3)
O3—C11—H11A	108.8	C8—S5—C9	102.3 (3)
C12—C11—H11A	108.8	C7—S6—C16	102.0 (3)
O3—C11—H11B	108.8		

### Hydrogen-bond geometry (Å, °)

**Table:**

*D*—H···*A*	*D*—H	H···*A*	*D*···*A*	*D*—H···*A*
C4—H4···O3^i^	0.93	2.35	3.127 (7)	141

Symmetry codes: (i) *x*−1/2, *y*+1/2, *z*.
